# Percutaneous endoscopic gastrojejunostomy for a patient with an intractable small bowel injury after repeat surgeries: a case report

**DOI:** 10.1186/1752-1947-5-55

**Published:** 2011-02-10

**Authors:** Masayasu Hara, Satoru Takayama, Hiromitsu Takeyama

**Affiliations:** 1 Department of Gastroenterological Surgery, Nagoya City University, 1 Kawasumi, Mizuho-cho, Mizuho-ku, Nagoya 467-8601, Japan

## Abstract

**Introduction:**

The management of intestinal injury can be challenging, because of the intractable nature of the condition. Surgical treatment for patients with severe adhesions sometimes results in further intestinal injury. We report a conservative management strategy using percutaneous endoscopic gastrojejunostomy for an intractable small bowel surgical injury after repeated surgeries.

**Case presentation:**

A 78-year-old Japanese woman had undergone several abdominal surgeries including urinary cystectomy for bladder cancer. After this operation, she developed peritonitis as a result of a small bowel perforation thought to be due to an injury sustained during the operation, with signs consistent with systemic inflammatory response syndrome: body temperature 38.5°C, heart rate 92 beats/minute, respiratory rate 23 breaths/minute, white blood cell count 11.7 × 10^9^/L (normal range 4-11 × 10^9^/μL). Two further surgical interventions failed to control the leak, and our patient's clinical condition and nutritional status continued to deteriorate. We then performed percutaneous endoscopic gastrojejunostomy, and continuous suction was applied as an alternative to a third surgical intervention. With this endoscopic intervention, the intestinal leak gradually closed and oral feeding became possible.

**Conclusion:**

We suggest that the technique of percutaneous endoscopic gastrojejunostomy combined with a somatostatin analog is a feasible alternative to surgical treatment for small bowel leakage, and is less invasive than a nasojejunal tube.

## Introduction

Repeated surgical operations are sometimes the cause of severe intestinal adhesions. Surgery for such patients requires a longer operating time for adhesiolysis, and sometimes causes further intestinal injury. Unfortunately, when the injury results in intestinal perforation, surgical treatment is usually necessary, unless minimal sepsis and good drainage is obtained; however, further surgery can in turn lead to more intestinal adhesion and further injury.

We report a case of repeated postoperative intestinal leakage in a patient with severe intestinal adhesions caused by several previous surgeries. Two separate operative procedures failed to seal the leakage and resulted in a paralytic and distended bowel condition. Finally, percutaneous endoscopic gastrojejunostomy (PEG-j) was used, which was effective in sealing the leakage and allowing recovery of normal bowel function. Our patient tolerated the PEG-j tube well with minimal effect on her daily functioning. We suggest that this technique is useful for drainage of intestinal fluid and decompression of the bowel until intestinal closure occurs spontaneously, and has minimal effect on patient comfort.

## Case presentation

**Figure 1:**
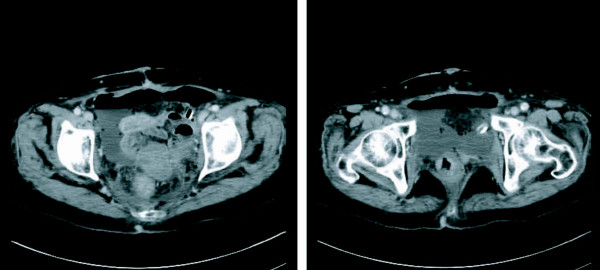
**Computed tomography images on postoperative day three**. Fluid collection and free air is visible at the previous surgical resection site.

A 78-year-old Japanese woman, who had undergone several laparotomies in the past, including an open drainage and sigmoidectomy because of peritonitis and colon cancer resection, underwent a curative bladder resection for bladder cancer via an extraperitoneal approach. On the third postoperative day (POD), a dirty brown discharge was noticed in a surgical drainage tube placed in the postbladder space, associated with a high fever and severe abdominal pain. Abdominal computed tomography (CT) showed fluid collection around a small bowel loop in the pelvis and in the upper abdomen (Figure 1). Urgent exploration through a midline incision revealed an injury 5 mm long in the small bowel injury at the base of the pelvis. Because of the presence of severe intestinal adhesions from the previous repeated surgeries, it as not possible to perform adequate bowel dissection for enterectomy and anastomosis, thus the intestinal injury was simply closed by a layer to layer suture.

After this second surgery, no fever elevation or discharge was noted, thus oral feeding was started on 11 days after the first surgery (eight days after the second), as abdominal radiography had not shown any evidence of obstruction or ileus. However, during that night, the patient had a sudden elevation in temperature and enteral drainage from the midline incision was seen. Computed tomography (CT) of the pelvis showed fluid collection and our patient was therefore prepared for further surgery. During the operation, adhesive bands between intestinal loops were dissected apart, the perforated bowel was removed, and intestinal continuity was reestablished via an end to end anastomosis. This operation took almost 10 hours, with estimated blood loss of 576 ml leading to marked tissue edema.

**Figure 2:**
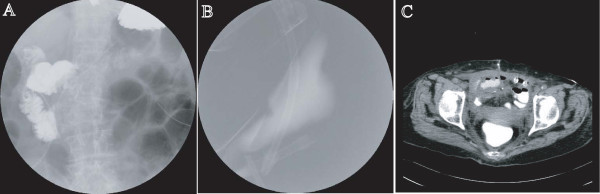
**Abdominal imaging eight days after the third operation**. **(A) **Both poor flow of contrast medium and intestinal distention are evident. **(B) **Radiograph of the drain bag demonstrating the presence of the contrast medium taken orally eight days after the third operation. **(C) **The day after this examination, fluid collection with extravasation of contrast medium is visible.

After this third operation, our patient's temperature was normal, but her small bowel was seen to be distended on abdominal radiographs. Contrast examination of the bowel performed on day 21 after the first surgery (day nine after the third surgery) revealed that the passage of contrast medium was poor, but it was unclear whether there was a leak (Figure 2A). After the examination, our patient experienced sudden abdominal pain and nausea. The following day, enteric drainage was again seen from the midline wound. Radiolography revealed that the contrast medium that had been administered orally the previous day was present in the extraperitoneal drain discharge (Figure 2B). CT also demonstrated the presence of extraluminal contrast medium (Figure 2C).

We considered it necessary to perform another intervention to close the intestinal injury; however, we concluded that a fourth surgery presented a high risk for this patient. Thus, we decided to treat her conservatively. For decompression and drainage of the intestine, a jejunostomy tube was thought to be necessary, and a percutaneous approach considered the best option. After we obtained our patient's informed consent, PEG-j tube (Transgastric Jejunal Catheter Kit with Funada style fixture; Create Medic Co. Ltd, Yokohama, Japan) was placed as described below.

**Figure 3:**
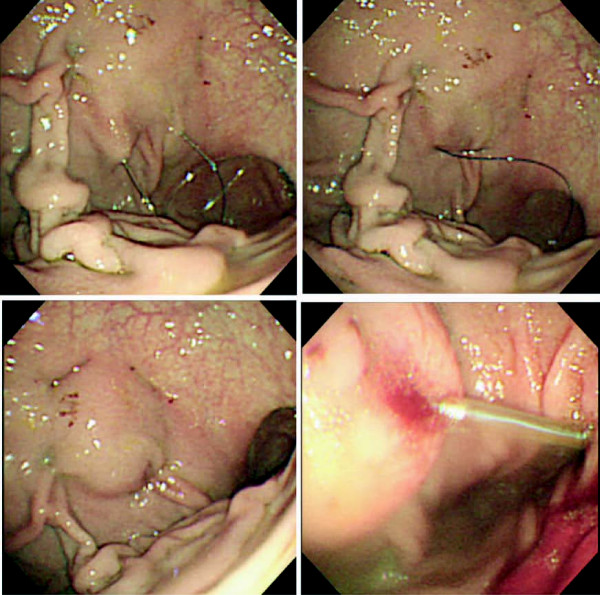
**Procedure for placing a percutaneous gastrojejunostomy**. Fixture with two puncture needles involving the abdominal and gastric wall, performed under endoscopic guidance. **(A) **The second needle is fed into the loop of a snare introduced into the gastric lumen through the first needle. **(B, C) **Four sutures are used around the insertion point. **(D) **Gastrojejunostomy tube inserted into the stomach and extended into the jejunum.

**Figure 4:**
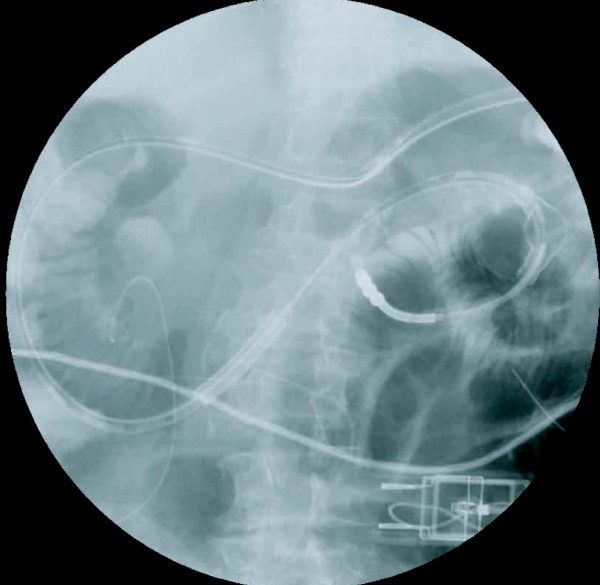
**Abdominal radiograph demonstrating percutaneous gastrojejunostomy tube placement**. The apex of the gastrojejunostomy tube was introduced into the upper jejunum 1100 mm from the insertion point. Severe intestinal distention can be seen.

Endoscopy was performed to identify a site of insertion for the tube by translumination and palpation of the abdominal wall. Under local anesthesia, the fixture was inserted into the stomach percutaneously via the anterior wall of the stomach. Through the first needle, an endoscopic snare was inserted into the gastric lumen. The suture was then fed out of the second needle into the loop (Figure 3A). The fixture was extracted and the suture ligated on the outside of the abdominal wall (Figure 3B). After raising the stomach to appose the abdominal wall, four sutures were placed around the site at which the gastrojejunostomy tube would be inserted (Figure 3B, C). A 16F enteric tube was inserted into the jejunum percutaneously (Figure 3D). Finally, the apex of the gastrojejunostomy tube was placed at the upper jejunum 1100 mm from the stomach (Figure 4). No complications or delayed wound infections were experienced.

**Figure 5:**
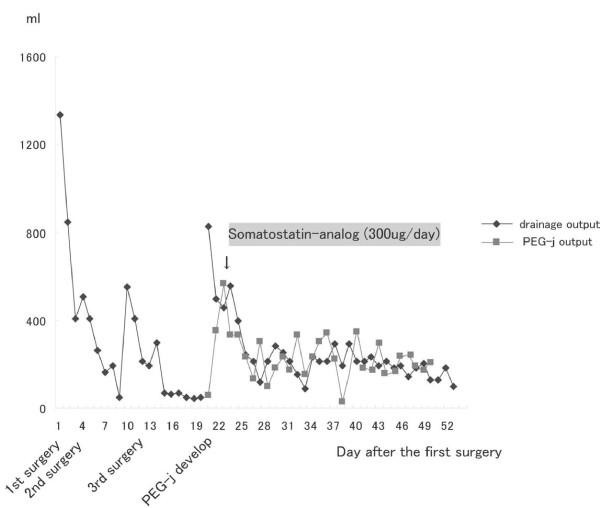
**Output from drain and percutaneous gastrojejunostomy tube after surgery**.

**Figure 6:**
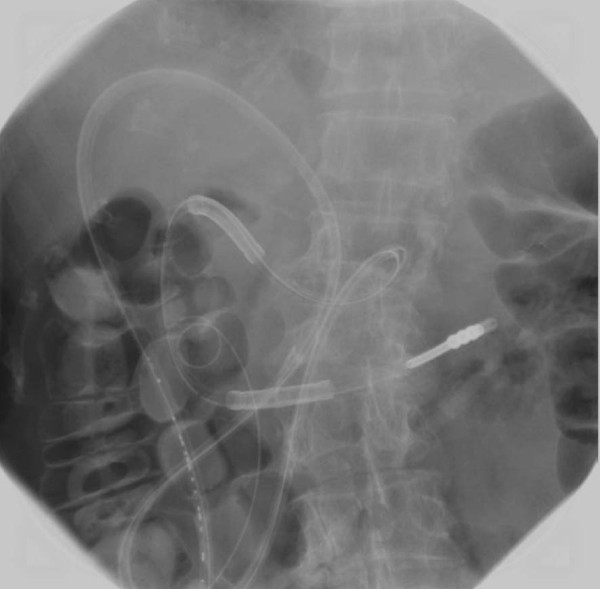
**Abdominal radiograph taken 28 days after gastrojejunostomy tube insertion**. Contrast medium administered via the tube reached the transverse colon. No obstruction or leakage was found. Intestinal distention was improved at this time.

A somatostatin analog was administered subcutaneously twice daily, and a proton pump inhibitor was administered intravenously once daily. The discharge from the gastrojejunostomy tube decreased dramatically from 500 ml to 120 ml per day (Figure 5), and amylase was not found in the abdominal drain. The PEG-j tube did not limit the activity of our patient. Radiological enteroclysis performed 22 days after the PEG-j tube placement showed not only an absence leakage but also recovery of intestinal flow and a normal gas pattern (Figure 6). After confirming that no leakage was present, oral feeding was started two days later (24 days after the PEG-j tube placement). After the PEG-j tube was removed, our patient was discharged, tolerating a regular diet and in good condition.

## Discussion

Repeated surgery can sometimes be the cause of intestinal adhesion and injury. Open laparotomy, especially for peritonitis, is one of the major causes of severe abdominal adhesions. Tough fibrous adhesions form between loops of bowel and the abdominal wall, or between individual bowel loops. This complicates any further surgery, because of the lengthy and difficult procedure of adhesiolysis required. This makes the surgery longer and can lead to greater blood loss than would ordinarily be experienced, making surgery more invasive and recovery of bowel function more delayed. When solid adhesions are present between bowel loops, these are easily injured. If an intraoperative intestinal injury has not been adequately repaired, an intestinal leak will occur. Once this has occurred, persistent inflammation and autodigestion by intestinal enzymes such as peptidase, saccharase and lipase retard bowel healing.

Thus, small bowel perforation is thought to be difficult to treat conservatively, and is usually regarded as a strong indication for further surgery. Even if surgical treatment is avoided with conservative therapy, enterocutaneous fistula (ECF) is often seen to develop. ECF is a difficult condition to cure, decreases the patient's quality of life, and can be a significant cause of mortality. Furthermore, portions of the intestine that undergo adhesiolysis often have a delay in recovery of peristalsis, which can lead to a rise in intraluminal pressure and result in anastomotic breakdown. Repeated surgery can often make the situation worse..

The repeated intestinal leakage in our patient was thought to have occurred as a result of intraoperative injury and rise in intraluminal pressure. We were concerned that a fourth surgery might lead to further adhesion and another intestinal injury that would make our patient's systemic and bowel condition worse. Thus, we decided to opt for a conservative management strategy instead of surgical treatment.

A number of conservative treatments for leakage and ECF have been reported, such as fibrin glue and VAC therapy [1-5]. In addition to these, we consider that jejunostomy tubes can be a useful conservative treatment for such intestinal disorders, because it allows effective drainage of intestinal content, which is a significant cause of delay in healing. However, it is usually placed via a nasal approach. Nasoenteral tubes are frequently left in place for a long time so that the tube becomes a source of harm for the patient, being the cause of aspiration pneumonitis and hampering patient activity. Thus, a percutaneous approach is more feasible and likely to be better tolerated than a nasal approach.

PEG or PEG-j has been mainly used for enteral nutrition therapy. The tubes are superior to nasojejunal tubes in terms of patient comfort and minimization of activity limitation, and carry almost no risk of aspiration pneumonia. There are some reports of their use as palliative therapy for decompression of malignant bowel obstruction [6-8]; however, to the best of our knowledge, there have been no reports about the utility of PEG-j for acute intestinal injury. In addition to its use as palliative therapy, we suggest that PEG-j is also useful for acute intestinal injury for which surgical treatment is not suitable, such as in our patient. In addition to this, the somatostatin analog we used has been reported to be useful in the management of intestinal leakage.

## Conclusion

PEG-j is a useful technique for managing acute intestinal injury for which surgical treatment is unsuitable.

## Competing interests

The authors declare that they have no completing interests.

## Consent

Written informed consent was obtained from the patient for publication of this case report and accompanying images. A copy of the written consent is available for review by the Editor-in-Chief of this journal.

## Authors' contributions

MH was a major contribution in writing the manuscript. ST developed PEG-j for the patient. All authors contributed to the patient's therapy. All authors read and approved the final manuscript.
